# A fast regulation algorithm for low voltage in regional grids with high uncertainty and high penetration of wind and solar loads at the grid end

**DOI:** 10.1371/journal.pone.0328057

**Published:** 2025-12-15

**Authors:** Heran Kang, Hongyang Liu, Lijun Zhao, Jie Sun, Yong Shi, Wei Yue, Miaoyong Feng

**Affiliations:** 1 Economic and Technological Research Institute, State Grid East Inner Mongolia Electric Power Co., Ltd., Hohhot, China; 2 South China University of Technology, Guangzhou, China; Aalto University, FINLAND

## Abstract

To address the issue of low voltage fluctuations at the grid end caused by the high penetration of wind and solar loads in the power grid, a fast low-voltage regulation algorithm is proposed. This algorithm aims to resolve the stability challenges brought by the uncertainty of wind and solar power sources. The method involves predicting the power output fluctuations of wind turbines, photovoltaics, and loads. A multi-objective fast regulation model is constructed, incorporating safety, efficiency, and cost factors, with corresponding constraints established. A simultaneous optimization transmission mechanism is adopted to achieve rapid voltage regulation. Results indicate that the algorithm has a high degree of prediction accuracy, rapidly restoring voltage to normal levels and maintaining stability within a tolerance level of 0.95 or higher. In conclusion, this algorithm effectively improves the stability and reliability of the power grid, providing a practical technical solution for handling high penetration levels of wind and solar energy in the grid.

## Nomenclature

*Abbreviations*

CGA: Co-evolutionary Genetic Algorithm
GFCs: Grid-Following Converters
OLTC: On-Load Tap Changer
LCC: Life Cycle Cost
PDF: Probability Density Function
EENS: Expected Energy Not Supplied
SOC: State of Charge
PV: Photovoltaic

*Sets, Parameters, indices, variables and functions*

*P*_*W*,*t*_
: The predicted wind power output at time t

v

The wind speed
*S*_*W*_
: The swept area of the wind turbine blades
*C*_*p*_
: The power coefficient
*ρ*
: The air density
*g*(*P*_*W*,*t*_): The probability density function of the wind turbine output power at time t

$\delta_{W,t}$

The standard deviation of the prediction error

$P_{pv,t}$

The PV power output at time t
*E*
: The irradiance

$S_{pv}$

The effective area of the PV array
*η*
: The efficiency of the PV system
*α*/*I*_*sc*_/*U*_*oc*_: Parameters related to PV cell characteristics

$g(P_{pv,t})$

The probability density function for the PV power output

$\delta_{pv,t}$

The standard deviation of the prediction error for PV power output

$g(P_{\text{load}})$

The probability density function of the load power
*P*_*A*_
: The mean load power

$\delta_{A}$

The standard deviation of the load power prediction error
*D* ^*c*^
: The total power shortage loss at the grid end
*f*_*s*_
: The evaluation rate for power shortage loss at the grid end
*EENS*_*k*_
: The expected energy not supplied (EENS) in the k-th year

$\mu_{\xi }$

The probability of occurrence of fault state
*n*
: The total number of nodes in the system
*X* ^*G*^
: The set of fault events in the grid

$Z_{i\xi }$

The amount of load shedding at node i under state ξ at the grid end
*R* ^*c*^
: The efficiency index
*T*
: The time period
$A_{LAk}^{c}$/$A_{LBk}^{c}$
The network loss values in different conditions for the k-th time step
$U_{2^{\prime }}$/$U_{3^{\prime }}$
The actual voltage deviations at two different nodes or points
$U_{2,\max}$/$U_{2,\min}$/$U_{3,\max}$/$U_{3,\min}$
The maximum and minimum voltage limits for nodes 2 and 3, respectively

$C_{I}^{c}$

The investment cost

$C_{O}^{c}$

The operating cost

$C_{F}^{c}$

The failure cost

$C_{M}^{c}$

The maintenance cost

$C_{D}^{c}$

The decommissioning and disposal cost
$D^{c}(x,y,z,w;\xi )$/$R^{c}(x,y,z,w;\xi )$/$C^{c}(x,y,z,w;\xi )$
The safety, efficiency, and cost indices, respectively

$I_{m,k}^{c}$

The current magnitude flowing through branch *m* in year *k*

$I_{m,\max}$

The maximum allowable current

$u_{h}^{c}$

The capacitor capacity

$u_{h,\max}$

The upper limit of capacitor capacity

$z_{k}^{c}$

The tap position of an OLTC transformer

$e_{g}^{c}$

The number of new substations

## 1 Introduction

Wind and solar energy, among other renewable energy sources, have developed rapidly. Due to their clean and renewable nature, these energy sources are widely regarded as essential components of the future energy structure. However, wind and solar power generation are influenced by natural conditions and exhibit significant intermittency and volatility. Variations in wind speed and solar irradiance can cause rapid fluctuations in power output, which can lead to instability in grid frequency, especially at the grid end in regional areas. This instability can exacerbate voltage drops, making it challenging to maintain stable voltage levels at the grid end.

Rapid voltage drops can lead to equipment damage or even cascading failures, affecting the overall stability of grid operations. Therefore, developing an efficient and precise fast regulation algorithm for low voltage at the grid end has become crucial to enhance the system’s adaptability and resilience.

To address the challenges posed by high wind and solar load penetration at the grid end, researchers have proposed various solutions, approaching the problem from perspectives such as modeling-driven predictions, control strategy optimization, flexibility analysis, and coordination among energy sources. For example, Chen Xijin et al. proposed a solution based on sensitivity analysis of fully embedded flexibility [1]. They first established a photovoltaic storage model and guided the power system’s sensitivity matrix, followed by clustering for optimization. By implementing an overall strategy with regional divisions, they optimized energy storage, wind, and photovoltaic output, achieving fast regulation of low voltage at the grid end. However, this study did not fully consider the impact of prediction errors in renewable energy sources on voltage stability. Zhang Z et al.’s study emphasizes the coordinated control of sources, grids, and loads [2]. This research proposes a coordinated control framework that reduces operating costs and distribution losses at the grid end through multi-objective optimization. By enabling fast coordination of inverters, energy storage devices, and reactive power compensation equipment, this framework addresses the low-voltage issues that arise when wind and solar loads are integrated into the grid. However, the study does not fully consider the impact of prediction errors in wind and solar output on the effectiveness of the coordinated control strategy. This oversight could result in situations where the control strategy fails to effectively manage the uncertainties in wind and solar output, thereby impacting voltage stability at the grid end. The study by Hao Jie et al. focuses on the conversion characteristics of wind and solar loads, establishing a steady-state model of the grid [3]. This model analyzes historical data to predict regions at the grid end that may experience low voltage, subsequently creating a multi-energy coordination strategy for fast low-voltage regulation. This strategy is formulated and solved to achieve quick low-voltage adjustment. However, this research relies heavily on historical data to predict potential low-voltage regions, which may not account for the real-time variability and uncertainty of wind and solar power output. Visser L et al.’s research centers on optimizing control strategies, particularly the GFCs dual-loop voltage-current control strategy [4]. By optimizing the current loop and introducing a discrete resonant controller to avoid the damping region, the study enhances the fast response of voltage regulation. However, this research places excessive focus on rapid response in control strategy, neglecting safety and economic factors in grid operation, especially the impact of wind and solar power output uncertainties on control effectiveness.

To address the aforementioned limitations, a fast low-voltage regulation algorithm is proposed. This algorithm not only considers the inherent volatility of wind and solar power output but also takes into account the uncertainties introduced by prediction errors, modeling this uncertainty as a key factor impacting voltage stability at the grid end. This approach provides a more comprehensive reflection of the complexities and uncertainties in actual grid operation, making the constructed model more realistic.

The low-voltage fast regulation model, built with this uncertainty in mind, incorporates multiple optimization objectives, including safety, efficiency, and cost, making the regulation strategy more balanced and holistic. By using a co-evolutionary genetic algorithm to solve this model, the algorithm can achieve fast and effective voltage regulation while ensuring the safe and stable operation of the grid, ultimately reducing economic costs.

## 2 Analysis of high penetration wind and solar load uncertainty in output

The output of wind and solar energy is influenced by numerous uncontrollable factors, such as weather conditions and equipment malfunctions, resulting in significant volatility and uncertainty in their power output. Analyzing this uncertain output provides a more accurate reflection of these influencing factors, leading to a more comprehensive understanding of the complexities faced in actual grid operations [5]. To maintain stable voltage levels at the grid end, it may be necessary to adjust the output of conventional generators, activate backup power sources, or use energy storage systems.

Therefore, to effectively address the uncertainty introduced by high penetration of wind and solar loads at the grid end, it is essential to establish more effective scheduling and control strategies for low voltage regulation at the grid end. This requires an analysis of the fluctuation characteristics of wind and photovoltaic (PV) power generation. Wind power tends to fluctuate more frequently and intensely, especially over short time intervals, while PV power fluctuations are generally smoother but are heavily influenced by weather changes. Analyzing these two types of fluctuations separately helps to develop targeted regulation strategies.

To thoroughly understand and quantify the uncertain output characteristics when a high proportion of wind and solar power is integrated into the grid, this section derives detailed output models for wind power, PV, and grid load. These models not only reflect the fundamental physical relationships of each output type (such as the relationship between wind speed and wind power output, and between irradiance and PV output) but also incorporate probability density functions for prediction errors to fully account for the uncertainties present in actual operations.

(1) Wind Power Output

By using Eq (1), a mathematical relationship is established between wind power output and wind speed. Additionally, a probability density function (PDF) for wind power output prediction error is introduced to quantify the impact of wind speed fluctuations on wind power output [6,7]. This approach enables a more accurate prediction of wind power output, providing a foundation for subsequent voltage regulation strategies.

$$\left\{ \begin{array}{c} P_{W,t}=\frac{1}{2}\rho S_{W}v^{3}C_{p}\\ g(P_{W,t})=\frac{1}{\sqrt{2\pi \delta_{W,t}}}e^{-\frac{P_{W,t}^{2}}{2(\delta_{W,t}{)}^{2}}} \end{array}\right.$$ (1)

In the formulation, *P*_*W*,*t*_ is the predicted wind power output at time t; *v* is the wind speed; *S*_*W*_ is the swept area of the wind turbine blades; *C*_*p*_ is the power coefficient; *ρ* is the air density; *g*(*P*_*W*,*t*_) is the probability density function of the wind turbine output power at time t; $\delta_{W,t}$ is the standard deviation of the prediction error for *P*_*W*,*t*_ following a normal distribution.

(2) Photovoltaic (PV) Power Output

Eq (2) describes the relationship between PV power output and irradiance, the effective area of the PV array, and factors such as the efficiency of PV conversion. It also considers the uncertainty caused by prediction errors in PV power output [8]. This helps in understanding the fluctuation characteristics of PV output and the impact of weather changes on the performance of PV systems.

$$\left\{ \begin{array}{c} P_{pv,t}=ES_{pv}\eta \\ \eta =\frac{\alpha \times I_{sc}\times U_{oc}}{E\times S_{pv}}\times 100\%\\ g(P_{pv,t})=\frac{1}{\sqrt{2\pi \delta_{pv,t}}}e^{-\frac{P_{pv,t}^{2}}{2(\delta_{pv,t}{)}^{2}}} \end{array}\right.$$ (2)

In the formulation, $P_{pv,t}$ is the PV power output at time t; *E* is the irradiance; $S_{pv}$ is the effective area of the PV array; *η* is the efficiency of the PV system; *α*, *I*_*sc*_ and *U*_*oc*_ are parameters related to PV cell characteristics; $g(P_{pv,t})$ is the probability density function for the PV power output; $\delta_{pv,t}$ represents the standard deviation of the prediction error for PV power output.

(3) Load Power Output

Using Eq (3), a probability density function for the active power of grid load is provided, reflecting the uncertainty in load demand. This uncertainty similarly affects the voltage stability of the grid, and therefore needs to be considered in voltage regulation strategies. The specific probability density function for load values is as follows:

$$g(P_{\text{load}})=\frac{1}{\sqrt{2\pi \delta_{A}}}e^{-\frac{(P_{\text{load}}-P_{A}{)}^{2}}{2\delta_{A}^{2}}}$$ (3)

In the formulation, $g(P_{\text{load}})$ is the probability density function of the load power; $P_{\text{load}}$ is the load power; *P*_*A*_ is the mean load power; $\delta_{A}$ is the standard deviation of the load power prediction error.

The focus of this research is on the uncertainties of these random variables, as they directly impact the voltage stability, frequency control, and power balance of the grid. By establishing probability density functions for these random variables, a better understanding and quantification of uncertainties within the grid can be achieved. This, in turn, enables the design of more effective control strategies and regulation measures to ensure the safe and stable operation of the grid.

Let *g*(*P*_*W*,*t*_), $g(P_{pv,t})$, and $g(P_{\text{load}})$ be the random variables for analyzing the uncertain output of high-penetration wind and solar power [9,10]. The proportional selection method is used to analyze the randomness of these variables. By normalizing the probabilities, an error interval is randomly selected and marked as the binary variable B, thereby obtaining the expression for the uncertain output of wind and solar power integration at the grid end.

$$\zeta =\left[B_{\left(1,t,s\right)}^{g(P_{W,t})},\ldots ,B_{\left(7,t,s\right)}^{g(P_{W,t})},B_{\left(1,t,s\right)}^{g(P_{pv,t})},\ldots ,B_{\left(7,t,s\right)}^{g(P_{pv,t})},B_{\left(1,t,s\right)}^{g(P_{\text{load}})},\ldots ,B_{\left(7,t,s\right)}^{g(P_{\text{load}})}\right]$$ (4)

In the formula, $B_{\left(i,t,s\right)}^{g(P_{W,t})}$, $B_{\left(i,t,s\right)}^{g(P_{pv,t})}$ and $B_{\left(i,t,s\right)}^{g(P_{\text{load}})}$ represent the binary control parameters for wind power generation, photovoltaic power generation, and power load demand at time t, respectively.

## 3 A fast regulation algorithm for low voltage

### 3.1 Establishment of low-voltage regulation model

The voltage at the grid end needs to be maintained within a certain range in real-time to ensure the stable operation of the power system. However, the randomness of wind, solar, and load generation makes real-time voltage stability more challenging. Therefore, a mechanism is needed to regulate voltage [11,12] to address this uncertainty.

To solve this problem, the output prediction errors of wind turbines and photovoltaic arrays, as well as load uncertainty, are taken into account. A target function is constructed that minimizes voltage deviation while also considering cost, efficiency, and feasibility. This algorithm enables the identification of the optimal voltage regulation strategy to ensure voltage stability at the grid end.

(1) Safety Index

By quantifying the total power shortage loss at the grid end, this index directly reflects the importance of voltage stability at the grid end. It emphasizes the necessity of regulating voltage to ensure the safe operation of the power system. Calculating the total power shortage loss at the grid end helps to enhance voltage stability, thereby ensuring the overall operational safety of the power system. The total power shortage loss at the grid end is given by:

$$\left\{ \begin{array}{c} D^{c}=\sum_{k=1}^{T}EENS_{k}\cdot f_{s}\\ EENS_{k}=\mu_{\xi }\cdot \sum_{i=1}^{n}Z_{i\xi },\;\xi \in X^{G} \end{array}\right.$$ (5)

In the formulation, *D*^*c*^ represents the total power shortage loss at the grid end; *f*_*s*_ is the evaluation rate for power shortage loss at the grid end; T denotes the planning period in years; *EENS*_*k*_ is the expected energy not supplied (EENS) in the k-th year; $\mu_{\xi }$ represents the probability of occurrence of fault state; *n* is the total number of nodes in the system; *X*^*G*^ is the set of fault events in the grid; $Z_{i\xi }$ is the amount of load shedding at node i under state ξ at the grid end [13].

(2) Efficiency Index

The calculation of the efficiency index considers the actual values of network loss and voltage deviation, factors that are directly related to the operational efficiency and stability of the power grid. Optimizing these indicators helps improve the overall efficiency of the grid. The formula for calculating the efficiency index is as follows:

$$R^{c}=\frac{\sum_{k=1}^{T}(A_{LAk}^{c}-A_{LBk}^{c})}{A_{LAk}^{c}}+\frac{U_{2,\max}-U_{2^{\prime }}}{U_{2,\max}-U_{2,\min}}+\frac{U_{3,\max}-U_{3^{\prime }}}{U_{3,\max}-U_{3,\min}}$$ (6)

In the formulation, *R*^*c*^ is the efficiency index; *T* represents the time period; $A_{LAk}^{c}$ and $A_{LBk}^{c}$ are the network loss values in different conditions for the k-th time step [14]; $U_{2^{\prime }}$ and $U_{3^{\prime }}$ are the actual voltage deviations at two different nodes or points; $U_{2,\max}$, $U_{2,\min}$, $U_{3,\max}$ and $U_{3,\min}$ represent the maximum and minimum voltage limits for nodes 2 and 3, respectively.

(3) Cost Index

The introduction of the cost index (Life Cycle Cost, LCC) ensures the economic feasibility of the regulation strategy. In addition to pursuing voltage stability and safe operation, considering cost-effectiveness is an important factor in practical engineering applications. The establishment of this index makes the proposed regulation algorithm more in line with practical requirements. The Life Cycle Cost (LCC) includes the investment cost $C_{I}^{c}$, operating cost $C_{O}^{c}$, failure cost $C_{F}^{c}$, maintenance cost $C_{M}^{c}$, and decommissioning and disposal cost $C_{D}^{c}$. The specific formula for the cost at the grid end is:

$$C^{c}=C_{I}^{c}+C_{O}^{c}+C_{F}^{c}+C_{M}^{c}+C_{D}^{c}$$ (7)

The objective function is based on safety, efficiency, and cost indicators, aiming to optimize the low-voltage regulation at the grid end following the integration of high-proportion wind and solar loads with significant uncertainty [15]. The decision variables are defined as follows: x for line upgrades, y for the number of reactive power compensation capacitors, z for transformer tap settings, and w for the number of new substations. During optimization, the output prediction errors of wind turbines and photovoltaic arrays, as well as load uncertainty, are considered. The objective function is formulated as follows:

$$G=\min\left[D^{c}(x,y,z,w;\xi ),R^{c}(x,y,z,w;\xi ),C^{c}(x,y,z,w;\xi )\right]$$ (8)

In the formulation, $D^{c}(x,y,z,w;\xi )$, $R^{c}(x,y,z,w;\xi )$ and $C^{c}(x,y,z,w;\xi )$ represent the safety, efficiency, and cost indices, respectively.

To restrict the range of decision variables (*x*, *y*, *z*, *w*) and maintain the operational status of the grid’s endpoints, the following constraints are established:

(1) Node Voltage Constraints:

Upper and lower bounds are set for node voltages, ensuring that:

$$B_{i,\min}\leq B_{i,t}^{c}\leq B_{i,\max}$$ (9)

(2) Branch Current Constraints:

For branch currents, let $I_{m,k}^{c}$ denote the current magnitude flowing through branch *m* in year *k*, and $I_{m,\max}$ represent the maximum allowable current. The constraint for branch current is defined as:

$$I_{m,k}^{c}<I_{m,\max}$$ (10)

(3) Reactive Power Compensation Capacitor Constraint:

For the switching capacity of reactive power compensation capacitors, let $u_{h}^{c}$ represent the capacitor capacity [16,17], with $u_{h,\max}$ as the upper limit. The constraint is defined as:

$$u_{h}^{c}<u_{h,\max}$$ (11)

(4) On-Load Tap Changer (OLTC) Transformer Tap Position Constraint:

For the tap position $z_{k}^{c}$ of an on-load tap changer (OLTC) transformer, set lower and upper limits, denoted as $z_{k,\min}$ and $z_{k,\max}$, respectively. The constraint is defined as:

$$z_{k,\min}<z_{k}^{c}<z_{k,\max}$$ (12)

(5) New Substation Quantity Constraint:

For the number of new substations, denoted as $e_{g}^{c}$, set an upper limit $e_{g,\max}^{c}$. The constraint is defined as:

$$e_{g}^{c}<e_{g,\max}^{c}$$ (13)

Based on the above-defined objective function and constraints, establishing the Low-Voltage Fast Regulation Model:

$$\left\{ \begin{array}{c} s.t.\max G(x;\xi )\leq g_{c}\\ g_{c}=(1+\epsilon )g_{x},\;x=1,2,\ldots ,\alpha \end{array}\right.$$ (14)

In the formulation, *β* represents the fluctuation amplitude of the uncertainty parameter; ξ is the input parameter; *x* denotes the decision variables of the objective function for low-voltage fast regulation; *g*_*c*_ represents the minimum expected target; *g*_*x*_ denote the solution set of the model.

### 3.2 Model solution

Under the conditions of high penetration of uncertain wind and solar energy in regional power grids, rapid fluctuations in power output may lead to power shortages, exacerbating voltage drops. The low-voltage regulation problem at the grid end involves multiple conflicting objectives, such as safety, efficiency, and cost. The Co-evolutionary Genetic Algorithm (CGA) can optimize these objectives simultaneously, finding a balance point to ensure safe grid operation while maximizing efficiency and minimizing costs [18]. Therefore, after defining the objective function and constraints of the low-voltage regulation model, the CGA algorithm is used to solve the model, achieving fast low-voltage regulation at the grid end under high penetration of uncertain wind and solar energy. The detailed solution process is as follows:

(1) Encoding Scheme

A mixed integer-real encoding scheme is used [19], in which the switching amount of reactive power compensation devices (integer variables) and the node voltages (real variables) are encoded. The switching amount of reactive power compensation devices is encoded as an integer sequence, with each integer value representing the switching state of different capacitors. The node voltages are encoded as a real number sequence, where each real value indicates the desired voltage level at the corresponding node.

(2) Population Initialization

Generate the initial population for the algorithm, which is a set of initial low-voltage regulation schemes for the grid end. Set the population size to N, meaning that there are N initial solution sets *g*_*x*_. Each solution set is converted into a format that the algorithm can process using the encoding scheme, and initial values are randomly generated to satisfy the constraint conditions.

(3) Evaluate Fitness Function

Calculate the fitness value of each individual to assess its quality. The fitness function is based on the objective function, with scenario probabilities used as weights. For individuals that do not meet the constraint conditions, a penalty function method is applied by adding a large penalty term to their fitness value. This ensures that these individuals are gradually eliminated during the genetic process.

$$\text{fitness}=\left\{ \begin{array}{c} C_{\max}-f,\;f<C_{\max}\\ 0,\;f\geq C_{\max} \end{array}\right.$$ (15)

In the formulation, *f* is the objective function value for the individual; $C_{\max}$ is the maximum allowable fitness threshold.

(4) Selection Operation

The selection operation combines the elitism strategy [20] with the roulette wheel selection method. First, the individual with the highest fitness in the current population is retained directly for the next generation. Then, the selection probability of each individual is calculated using the roulette wheel selection formula (16). Based on these probabilities, several individuals are randomly chosen from the remaining population to enter the next generation.

$$d_{i}=\text{fitness}[\max G(x;\xi )]$$ (16)

(5) Crossover Operation

Set the initial crossover probability *D*_*c*0_, the minimum crossover probability $D_{c\min}$, and the crossover probability reduction step $D_{c\text{step}}$. In each iteration, the crossover probability is dynamically adjusted based on the current generation and the crossover probability calculation formula. Two parent individuals are randomly selected for crossover. Depending on the variable type (integer or real), either arithmetic crossover or single-point crossover is performed to generate new offspring. The crossover calculation formula is as follows:

$$D_{c}=d_{i}\cdot (D_{c0}-D_{c\text{step}}\times \theta ),\;D_{c}<D_{c\min}$$ (17)

In the formulation, *θ* represents the genetic evolution generation (or generation number) in the genetic algorithm.

(6) Mutation Operation

Set the initial mutation rate *D*_*m*0_, maximum mutation rate $D_{\max}$, and mutation rate increment step $D_{m\text{step}}$. In each iteration, the mutation rate is dynamically adjusted based on the current generation number and the mutation rate calculation formula [21].

$$D_{m}=D_{m0}+D_{m\text{step}}\times \theta ,\;D_{m}<D_{\max}$$ (18)

Randomly select several individuals for mutation. For real-valued variables, use a small mutation strategy as shown in formula (19) to make fine adjustments, thereby increasing the diversity of the population.

$$D_{i}=(X_{i_{\max}}-X_{i_{\min}})\times \text{rand},\;\text{rand}<D_{m}\cdot D_{c}$$ (19)

In the formulation, $X_{i_{\max}}$ and $X_{i_{\min}}$ are the maximum and minimum bounds for the variable *X*_*i*_; rand is a random number between 0 and 1.

(7) Co-evolution and Power Flow Calculation

Distribute the low-voltage regulation variables of the power grid across multiple populations, where each population evolves independently but periodically exchanges optimal solution information. The co-evolution mechanism facilitates information sharing and strategy integration among populations, enhancing the global search capability. In each iteration, perform a power flow calculation on the selected populations to verify the effectiveness of the regulation schemes and update the fitness values accordingly.

(8) Iteration and Termination Conditions

Set the maximum genetic generation $\theta_{\max}$ as the termination condition for the algorithm. After each iteration, check if the fitness value of the best individual meets the preset convergence criteria or if the maximum genetic generation $\theta_{\max}$ has been reached. If either condition is satisfied, terminate the algorithm and output the optimal solution along with the corresponding values of the regulation variables.

Through the detailed solution process and parameter settings outlined above, the CGA algorithm is ensured to have greater practical guidance value and operability when solving the low-voltage fast regulation problem at the grid end under conditions of high penetration and uncertainty of wind and solar power integration.

### 3.3 Static Voltage Stability Margin (SVSM) calculation method

The static voltage stability margin (SVSM) is a critical indicator to quantify the distance between the current operating point and the voltage collapse boundary. In this study, SVSM is calculated based on P-V curve analysis following the IEEE 3002.2-2018 standard, combined with real-time data from Phasor Measurement Units (PMUs). The detailed steps are as follows:

Step 1: P-V Curve Construction

The maximum loadability limit ($P_{\max}$) is determined by incrementally increasing the active power load ($P_{\text{load}}$) at the target node until the system reaches the voltage collapse point. This process is implemented via continuation power flow (CPF) analysis, which iteratively solves the power flow equations while adjusting the load scaling factor (*λ*):

$$\lambda \cdot P_{\text{load}}+j\lambda \cdot Q_{\text{load}}=f(V,\theta )$$ (20)

Where *V* and *θ* are the voltage magnitude and phase angle, respectively.

Step 2: Real-Time Operating Power Acquisition

The actual operating power ($P_{\text{operating}}$) is obtained from PMUs installed at key grid nodes, providing synchronized measurements with a sampling rate of 30 frames per second.

Step 3: SVSM Calculation

The SVSM is defined as the normalized difference between $P_{\max}$ and $P_{\text{operating}}$:

$$\text{SVSM}=\frac{P_{\max}-P_{\text{operating}}}{P_{\max}}$$ (21)

A higher SVSM value (closer to 1) indicates a more stable system.

## 4 A fast regulation algorithm for low voltage

To validate the overall effectiveness of the rapid low-voltage regulation algorithm for power grids with a high proportion of uncertain wind and photovoltaic (PV) load integration at the grid’s end, it is necessary to conduct comprehensive testing.

### 4.1 Experimental preparation

The power system simulation software (SimPowerSystems in Simulink) is used to establish the grid model, perform simulation operations, and analyze results. A looped grid at the end of an actual power system in the Guangzhou area was selected as a case study. The grid data was obtained from the local utility company’s extensive historical records, covering a five-year period and encompassing critical parameters such as wind speed, solar irradiance, and load demand.

**Fig 1:**
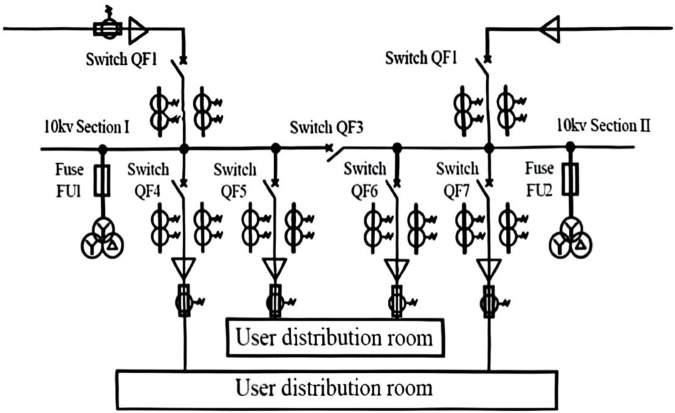
Block diagram of ring power grid.

Specifically, wind speeds ranged from 3 m/s to 15 m/s, with an average of 8 m/s. Solar irradiance data included both sunlight duration and intensity, ranging from 100 W/m^2^ to 1000 W/m^2^. Load demand statistics provided daily load curves, with a peak value of 600 MW and a trough of 300 MW. These data were used for model construction and simulation analysis to assess grid performance and serve as the foundation for algorithm design and validation. A block diagram of the looped grid is illustrated in Fig 1.

In the actual simulation model, a total of 80 nodes are set up within the system. These nodes are distributed according to the actual layout of the power grid, covering key elements within the study area, including power plants, substations, transmission lines, and load points. The node distribution takes into account factors such as the grid’s geographical characteristics, load density, power source locations, and transmission line corridors. This approach ensures that the simulation model accurately reflects the operational state of the power grid.

In the simulation model, the equipment parameters are configured as follows:

(1) The wind turbine has a cut-in wind speed of 4 m/s, below which the turbine ceases operation, and a cut-out wind speed of 26 m/s, above which it shuts down for safety. The rated wind speed is set at 13.5 m/s, where the turbine achieves optimal efficiency. The rated capacity is 400 kW, representing the maximum power output under standard conditions.

(2) The photovoltaic (PV) panels are configured with an operating voltage of 38 V to ensure optimal performance, and an operating current of 9.12 A, indicating the current output under the given conditions. The rated power is 350 kW, reflecting the maximum output under standard test conditions, and a conversion efficiency of 21%, indicating a high efficiency in converting solar energy to electrical power.

(3) The energy storage battery is set with a State of Charge (SOC) range between 10% and 90% to optimize lifespan and performance. The rated voltage is 3V, ensuring operation within standard voltage limits, with a rated capacity of 550 Ah representing its storage capability, and a charge/discharge efficiency of 90%.

The parameters for the wind power, photovoltaic, and load models, such as the Weibull distribution parameters and photovoltaic conversion efficiency, are set as shown in Table 1.

**Table 1:** Model parameter settings.

Model	parameters
Weibull distribution model	Scale parameter 15.0 m/s; Shape parameter 3.0;
Photovoltaic model	Photoelectric conversion efficiency of 15%; The effective area of the photovoltaic array is 5000 m^2^
Load model	Average load of 500 MW; Load standard deviation 50 MW

In the experiment, to ensure the effectiveness and reliability of the model solution, key parameters were configured and optimized. The population size was set to 100, with a maximum of 200 genetic generations. The crossover probability was initially set to 0.8 and gradually decreased to 0.5, while the mutation probability was initially set to 0.1 and gradually increased to 0.3. The weights for the fitness function were assigned as 0.4, 0.3, and 0.3 for safety, efficiency, and cost indices, respectively. These parameter settings enable effective resolution of the rapid low-voltage regulation model at the grid’s end under high levels of wind and photovoltaic uncertainty, yielding accurate simulation results.

### 4.2 Experimental procedure and result analysis

#### 4.2.1 Experimental procedure and result analysis.

A precise power grid model was constructed using power system simulation software, encompassing grid topology, component parameters, operational constraints, and the configuration of wind and photovoltaic (PV) integration points. Based on historical data and statistical analysis, a variety of wind-PV-load fluctuation scenarios were defined to comprehensively simulate uncertainties that may be encountered in real grid operations. These scenarios range from extreme high wind speed with low irradiance and high load to low wind speed with low irradiance and low load.

**Fig 2:**
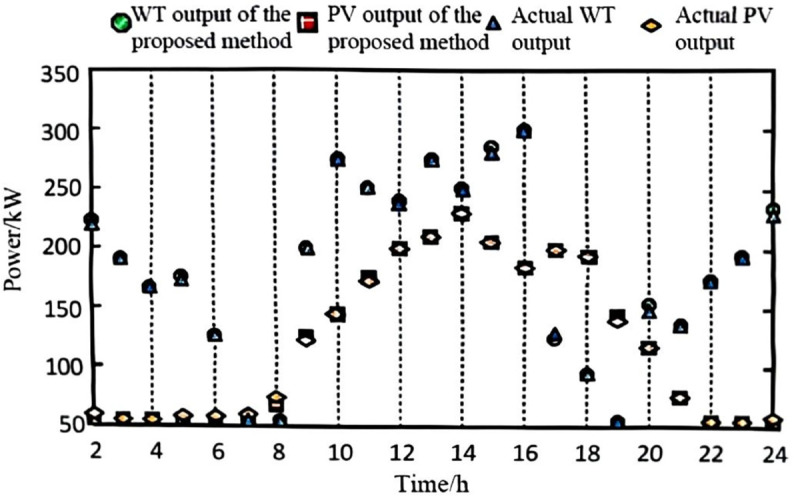
Wind power and wind power forecast output with the proposed algorithm.

**Fig 3:**
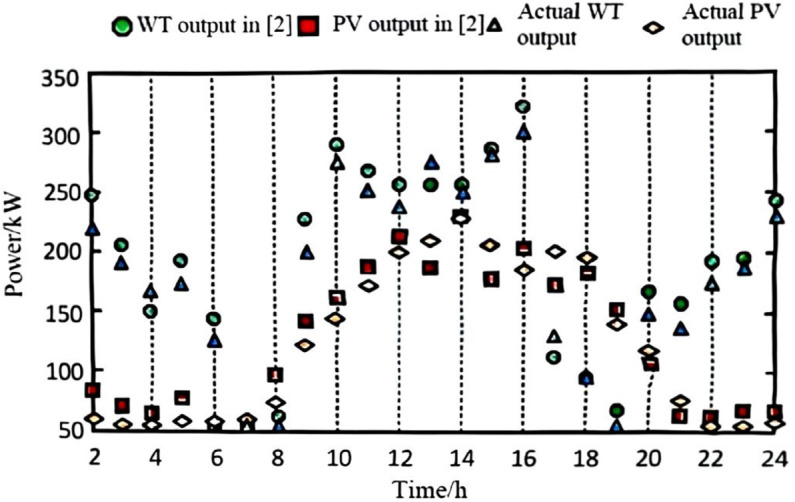
Wind power and wind power forecast output with method in [2].

**Fig 4:**
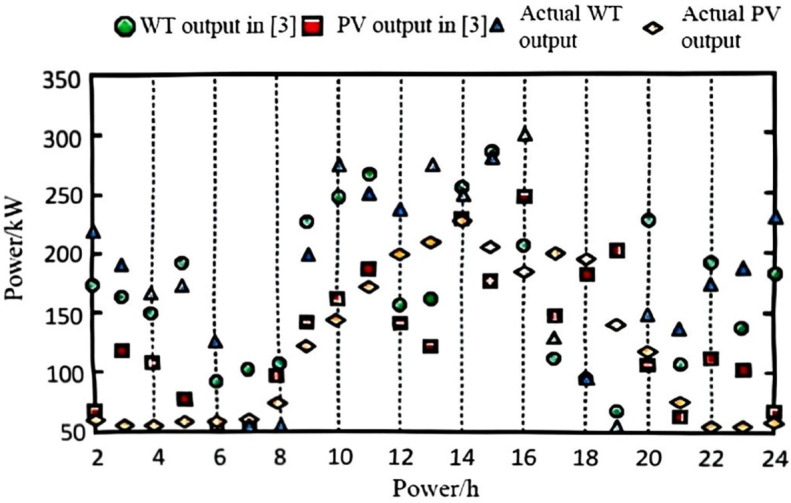
Wind power and wind power forecast output with method in [3].

**Fig 5:**
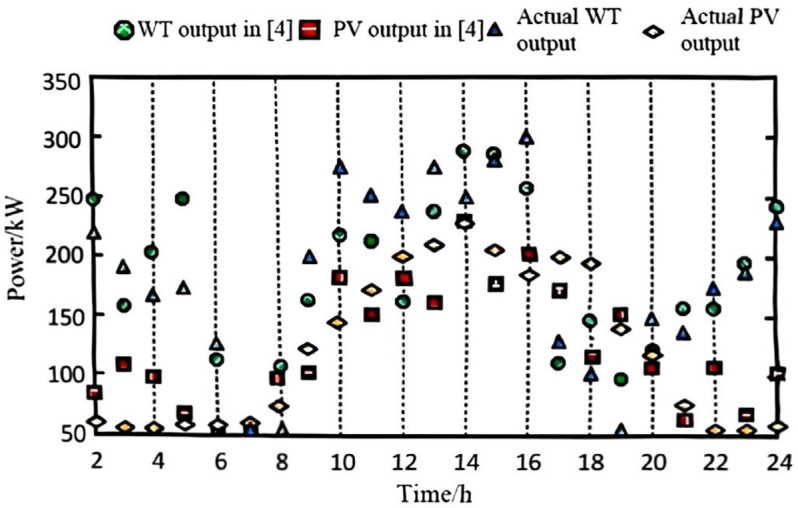
Wind power and wind power forecast output with method in [4].

The collected data and defined scenarios were input into the model, with the wind and photovoltaic (PV) data from the highest-probability scenario selected for input into the power system. The power values were obtained through a process of gathering and pre-processing historical data from wind farms, PV stations, and grid loads, followed by defining and selecting the highest-probability wind-PV-load fluctuation scenario. A forecasting algorithm was applied to account for power output prediction errors, and the predicted data were then input into the grid model for simulation. For comparison, the methods from references [2–4] were employed. The final predicted output for wind and PV power is shown in Figs 2, 3, 4, and 5.

As observed from the results in Figs 2, 3, 4, and 5, the proposed algorithm accurately predicts wind and photovoltaic (PV) outputs across various wind-PV-load scenarios. The probability distribution of each scenario is reasonable, and the predicted wind and PV output closely aligns with actual data. This high accuracy in predicting wind and PV output is due to the algorithm’s consideration of prediction errors for the output power of wind turbines, PV systems, and grid load as random variables, better reflecting real-world system uncertainties.

To quantitatively evaluate the prediction accuracy of the proposed algorithm, we compared its performance with methods in [2–4]. The Mean Absolute Error (MAE) and Root Mean Square Error (RMSE) were adopted as evaluation metrics. The results are summarized in Table 2.

**Table 2:** Comparison of prediction accuracy between the proposed algorithm and existing methods.

Method	MAE (MW)	RMSE (MW)
Proposed Algorithm	12.3	18.7
Method in [2]	15.8	22.4
Method in [3]	19.2	25.6
Method in [4]	13.5	20.1

As shown in Table 2, the proposed algorithm achieves the lowest MAE (12.3 MW) and RMSE (18.7 MW), outperforming other methods by 22.2% and 16.5%, respectively. This demonstrates its superiority in handling prediction uncertainties caused by high-penetration renewable energy integration.

This high accuracy in predicting wind and PV output is due to the algorithm’s consideration of prediction errors for the output power of wind turbines, PV systems, and grid load as random variables, better reflecting real-world system uncertainties. The incorporation of these random variables enables the model to account for output fluctuations across different scenarios, thereby enhancing prediction accuracy. This demonstrates the algorithm’s accuracy and effectiveness in handling uncertainty, providing a robust data foundation for subsequent low-voltage regulation strategies.

#### 4.2.2 Comparison of low voltage regulation performance.

**Fig 6:**
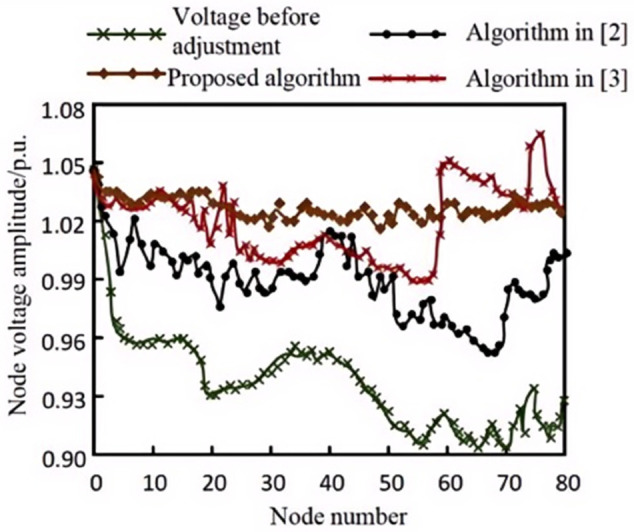
Low voltage regulation effect of each algorithm.

In studying the voltage drop at end nodes caused by fluctuations in uncertain wind and solar power outputs and load increases within a power grid region, the proposed algorithm was applied, along with the steady-state model-based approach from [3] and the dual-loop voltage-current control algorithm based on GFCs presented in [4], to regulate the voltage in this area. The voltage data at each node, recorded before and after the application of each algorithm, are presented in Fig 6 as a statistical summary.

Analysis of Fig 6 indicates that when voltage decreases due to fluctuations in uncertain wind and solar output and increases in load, the proposed algorithm can rapidly and effectively restore node voltage to normal or near-normal levels. This effectiveness is largely attributed to the algorithm’s consideration of the uncertainties in renewable energy output and forecast errors, which form the basis of an optimized model that can more accurately predict and address potential low-voltage conditions at grid endpoints. In contrast, the algorithm in [3] appears to have a slower regulation response, failing to restore voltage to normal levels within a short time. While the algorithm in [4] does achieve some degree of regulation at certain nodes, its overall performance is less pronounced than that of the proposed algorithm. Therefore, in practical applications where fast response and precise regulation are essential, the proposed algorithm stands as a more favorable choice.

#### 4.2.3 Comparison of static voltage stability margin.

Static voltage stability margin is a critical indicator for assessing a power system’s ability to maintain voltage stability following disturbances. In complex scenarios where a high proportion of wind and solar generation is integrated at the grid’s end nodes, accurately evaluating the effectiveness of various algorithms in enhancing voltage stability margin becomes particularly important. To this end, a diverse set of test scenarios was designed, and the performances of the proposed algorithm, the algorithm from [3], and the algorithm from [4] were meticulously recorded in these scenarios.

Under conditions of high uncertainty with a large proportion of wind and solar power integrated into the region, the following experimental tests were conducted. Based on historical data, scenario analysis was employed to define 15 wind-solar-load fluctuation scenarios, among which three of the most common scenarios are as follows:

Scenario 1: High wind speed, low solar irradiance, high load

Wind speed: 15 m/s; Solar irradiance: 200 W/m^2^; Load: 600 MW (peak period, above average)

Scenario 2: Low wind speed, high solar irradiance, medium load

Wind speed: 5 m/s; Solar irradiance: 1000 W/m^2^; Load: 450 MW (near average)

Scenario 3: Low wind speed, low solar irradiance, low load

Wind speed: 3 m/s; Solar irradiance: 100 W/m^2^; Load: 300 MW (below average)

**Fig 7:**
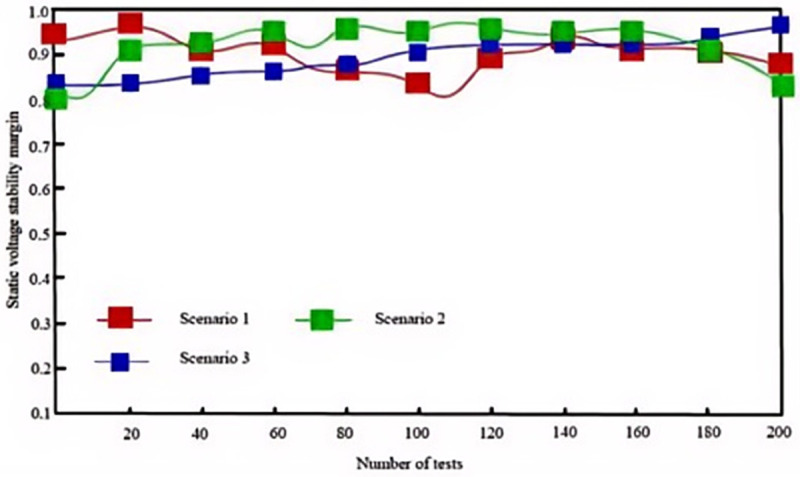
Static voltage stability margins with the proposed algorithm.

**Fig 8:**
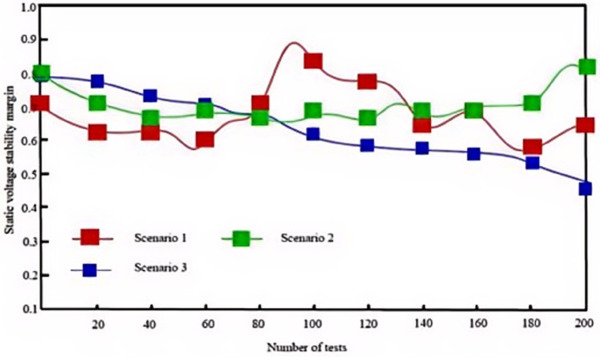
Static voltage stability margins with method in [3].

**Fig 9:**
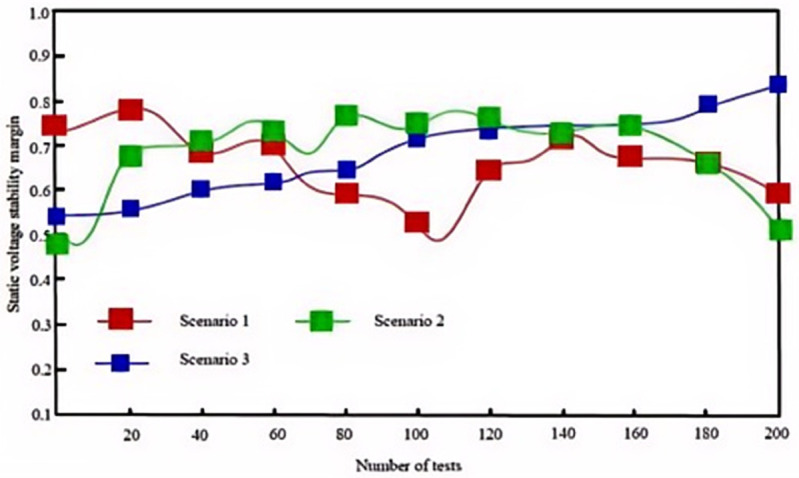
Static voltage stability margins with method in [4].

These three test scenarios encompass varying proportions of wind and solar integration, diverse load distribution patterns, and different network topology structures. In these complex and realistic scenarios, each of the three algorithms was applied for 200 dynamic adjustment operations. Changes in the static voltage stability margin of the system after each adjustment were recorded, and the statistical results are illustrated in Figs 7, 8, and 9.

Table 3 compares SVSM values across methods. The proposed algorithm achieves 0.82 in Scenario 1, 0.96 in Scenario 2, and 0.98 in Scenario 3, outperforming [3,4]. Notably, in Scenario 3, the algorithm maintains near-ideal stability (SVSM ≈ 0.98), demonstrating exceptional adaptability to low-demand conditions.

**Table 3:** Comparison of SVSM in different scenarios.

Method	Scenario 1	Scenario 2	Scenario 3
Proposed Algorithm	0.82	0.96	0.98
Method in [2]	0.65	0.78	0.82
Method in [3]	0.71	0.85	0.88

This indicates that the proposed algorithm is particularly effective in managing voltage fluctuations and instability resulting from high proportions of wind and solar power integration at the grid’s endpoints. This superior performance is attributed to the use of the CGA (Canonical Genetic Algorithm) within the algorithm, which does not rely on the selection of an initial solution during the search process. Consequently, even when grid conditions vary across different scenarios, the CGA can identify effective voltage regulation strategies, thereby maintaining a high voltage stability margin.

In contrast, although the algorithms in [3,4] exhibit a certain level of voltage stability in specific scenarios, their overall effectiveness is noticeably less pronounced compared to the proposed algorithm.

## 5 Conclusion

To address the uncertainties introduced by the high penetration of wind and solar generation at the grid’s endpoints, this study proposes an innovative rapid low-voltage regulation algorithm that significantly enhances the intelligence of grid scheduling and control. By thoroughly analyzing voltage fluctuation issues caused by the intermittent and uncertain nature of wind and solar energy, the algorithm constructs a stochastic variable model based on forecast error, accurately capturing the uncertainties of wind-solar-load integration. Building on this, the algorithm further designs a rapid low-voltage regulation model that integrates multiple objectives, including security, efficiency, and cost-effectiveness. This model is then precisely regulated through a co-evolutionary genetic algorithm to address low-voltage challenges at the grid’s endpoints.

Experimental results demonstrate that the algorithm effectively predicts wind and photovoltaic outputs, quickly restores voltage to normal or near-normal levels, and maintains a voltage stability margin above 0.95. This performance significantly enhances grid stability and reliability. Consequently, the proposed algorithm not only addresses limitations of traditional methods but also provides robust support for intelligent grid regulation, holding substantial practical application value and promising market potential.

## Supporting information

S1 Data (ZIP)
